# Steroid Dermatitis Resembling Rosacea: A Clinical Evaluation of 75 Patients

**DOI:** 10.1155/2013/491376

**Published:** 2013-04-21

**Authors:** Ammar F. Hameed

**Affiliations:** Department of Dermatology & Venereology, College of Medicine, University of Baghdad, Medical Collection Office, P.O. Box 61106, Baghdad 12114, Iraq

## Abstract

*Background*. The use of topical steroids on the skin of the face should be carefully evaluated by the dermatologist; however, its misuse still occurs producing dermatological problem resembling rosacea. *Objectives*. To report the different clinical manifestations of steroid dermatitis resembling rosacea and to discover causes behind abusing topical steroids on the face. *Methods*. In this prospective observational study, 75 patients with steroid dermatitis resembling rosacea who had history of topical steroid use on their faces for at least 1–3 months were evaluated at the Department of Dermatology, Baghdad Teaching Hospital, between August 2010 and December 2012. *Results*. The majority of patients were young women who used a combinations of potent and very potent topical steroid for average period of 0.25–10 years. Facial redness and hotness, telangiectasia, and rebound phenomenon with papulopustular eruption were the main clinical presentations. The most common causes of using topical steroid on the face were pigmentary problems and acne through recommendations from nonmedical personnel. *Conclusion*. Topical steroid should not be used on the face unless it is under strict dermatological supervision.

## 1. Introduction

Topical corticosteroids (TCS) are of great value in treating a wide spectrum of dermatological diseases and since the time of its introduction in 1951, a new therapeutic era in dermatology has been emerged [1].

The development of super potent corticosteroid in 1974 added more cutaneous diseases to the list of TCS indications. Meanwhile TCS misuse also appeared as a common problem adding a new complication which has been reported by variety of investigators [2]. Chronic misuse of TCS on the face produced a clinical condition which was described by various names, like light sensitive seborrheid [2], perioral dermatitis [3], rosacea-like dermatitis [4]. steroid rosacea [5], steroid dermatitis resembling rosacea [6], and steroid-induced rosacea-like dermatitis [7]. Since there is no agreement on nomenclature, we prefer to promote the term steroid dermatitis resembling rosacea (SDRR) where it describes the morphology of the disease due to TCS abuse on the face. The main clinical presentation of this dermatosis is diffuse facial redness with or without papulopustular lesions in addition to the development of rebound phenomenon after withdrawal of TCS [6].

This dermatosis is commonly seen in the daily clinical practice, but there are few reports describing it in the medical literature [6, 8]. 

The aim of the present study is to document the different clinical presentations of SDRR and to evaluate the purpose behind misusing TCS on the face. 

## 2. Patients and Methods

This descriptive case series study involved a total of 75 patients diagnosed as SDRR who consulted the Department of Dermatology and Venereology, Baghdad Teaching Hospital, between August 2010 and December 2012.

Inclusion criteria for patients to be enrolled in this study were the following.

Patients with clinical symptoms and signs suggestive of SDRR who had history of TCS use on the face continuously (for more than 1 month) or intermittently (for more than 3 months) due to any purpose other than classical rosacea.

Patients with natural rosacea or those denying any history of TCS on the face and pregnant women were excluded.

The diagnosis was established on clinical basis. A special questionnaire was designated to include all clinical data like demographics, age of patient at onset of the disease, duration of the disease, symptoms, and signs of the disease.

A particular attention was given to corticosteroid therapy regarding the type, potency, duration of therapy, purpose, and the source of its use.

Medical photographic documentation of the patients was done using Nikon COOLPIX 8000 camera. Formal consent was obtained from each patient after full explanation of the goals and the nature of the study to them and the study was approved by the Ethical Committee of College of Medicine, University of Baghdad. Descriptive statistical analysis was done by using scientific calculator. 

## 3. Results

Seventy-five patients with SDRR were evaluated. Their ages ranged between 18 and 60 years with a mean age ± SD of 29.6 ± 8 years. The mean duration of their TCS use was 3.5 ± 3 years with a range of 0.25–10 years. The female to male ratio was 4.7 : 1 (62 women versus 13 men). The main age groups affected are those between age of 21 and 30 (represent 53% of the affected group) and 31 and 40 (represent 33% of the patients) as seen in Table 1. The most frequently used fluorinated TCS were Betamethasone valerate 0.1 in 5 (6%) patients and Clobetasol propionate 0.05 in 7 (9%) patients or both of them in 15 (20%) patients while 48 (64%) patients had combined any one of the above-mentioned TCS with the available over-the-counter cosmeceuticals (the so-called fairness mixtures) as shown in Table 2.

The minimum duration needed to develop the SDRR was 3 months while the maximum duration was 10 years.

The main sources of TCS prescription were beauty centers (26 patients), self-prescription (20 patients) and Pharmacy advice (18 patients) as illustrated in Table 3.

The purposes behind TCS use were melasma in 25 (33%) patients, acne in 9 (12%) patients, freckles in 5 (6%), and actinic lichen planus in 2 (2%) patients while 31 (41%) patients used TCS as a fairness cream as outlined in Table 4.

Tables 5 and 6 show the clinical presentation and the triggering factors in patients with SDRR. 

## 4. Discussion

Corticosteroids are not the panacea for all forms of dermatological diseases but it is extremely valuable when their limitations are realized. TCS are the treatment of choice for a variety of cutaneous disorders when it is used on the appropriate site and in proper concentration. However, TCS should not be used on the face except for acute inflammatory conditions provided that it will be not used for more than one month [9, 10].

Previous reports and the present study have demonstrated that the chronic TCS use on the face could occur [11]. The uncontrolled prescriptions of TCS can be started by beauticians, chemists, and self-prescription or even by the dermatologist (Table 3).

The most commonly used preparations were fluorinated TCS including Betamethasone valerate 0.1, Clobetasol propionate 0.05, and sometimes in combination with other cosmeceuticals (Table 2). The authorization for fluorinated TCS prescription must be restricted only for the licensed dermatologist and the easy intake from pharmacies should be controlled.

Pigmentary problems like melasma, freckles, and actinic lichen planus and searching to have fairer look are the main motivators for the clients to use TCS on their faces (Table 4). Saraswat et al. reported that steroid combinations (mostly of potent and very potent TCS) are the most commonly abused preparations on the face where the most common indication was to achieve fairness. A nonphysician recommendation was documented in 59% of their cohort [12]. 

The pharmacological properties of steroid like the anti-inflammatory and vasoconstrictive effects are responsible for its dramatic effects on suppressing whatever the initial primary dermatosis and this will encourage the patients to continue on the TCS use without the supervision of the medical authorities.

**Figure 1:**
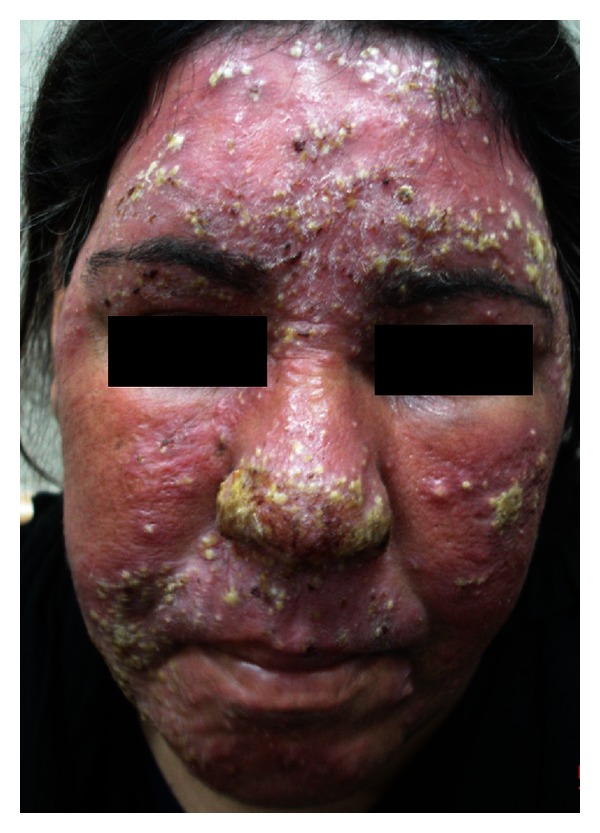
Rebound phenomenon in form of diffuse papulopustular eruption in a young woman with SDRR after TCS withdrawal.

**Figure 2:**
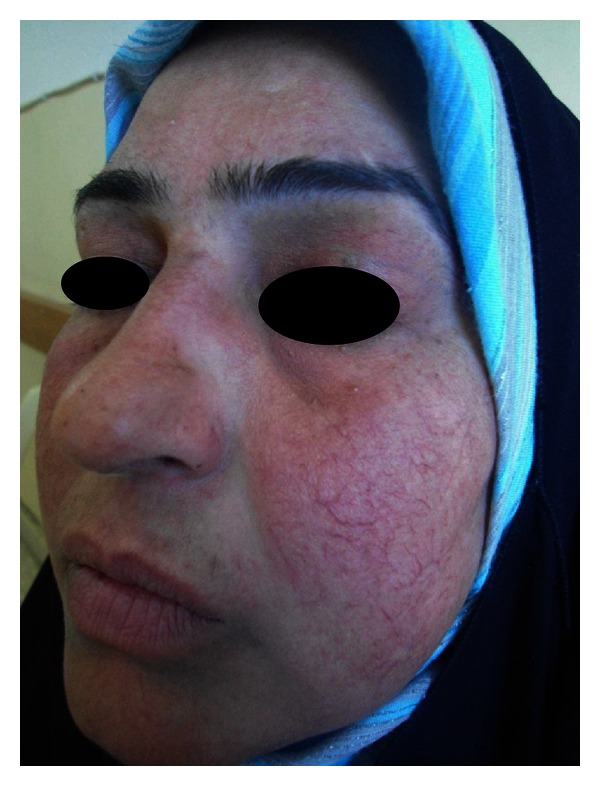
Prominent telangiectasia with background of diffuse erythema in SDRR patient presented with hot flushing.

Upon withdrawal of the therapy, recurrence will occur and repeated cycles of relapse and remission will start [11]. Development of tachyphylaxis necessities increment in dosage and furthermore the emergence of side effects of TCS including diffuse redness, papulopustular eruption (Figure 1), telangiectasia (Figure 2), dry skin, and rebound phenomenon which all represent the main clinical features of SDRR [13] (Table 5). Most patients had history of exacerbation of the symptoms and signs of SDRR after heat exposure, emotional stress, hair epilation (threading), and sun exposure but not hot drinks (Table 6).

**Figure 3:**
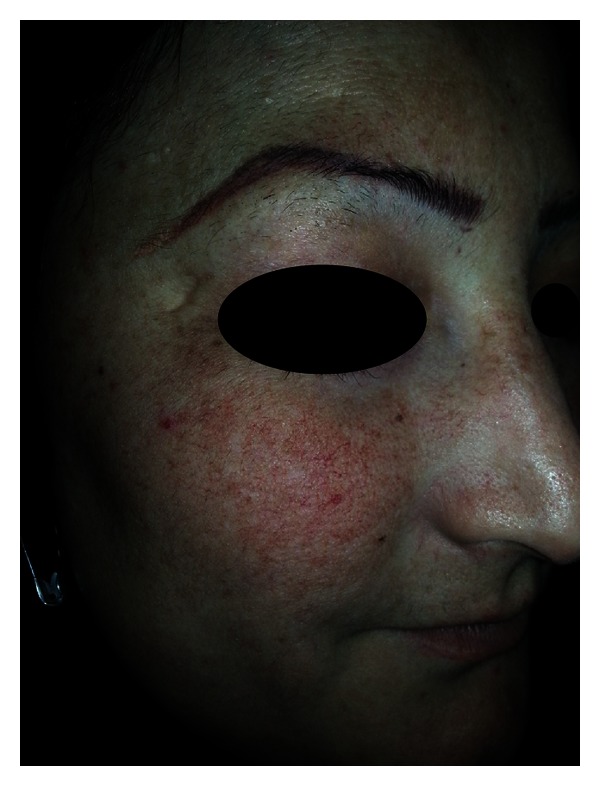
Self-prescription of potent TCS for 1 year duration in young women with melasma resulted in development of SDRR.

Young women are the most affected population by this problem (Table 1). This can be partly explained by the higher prevalence of pigmentery disorders like melasma in females and the fact that women always respond promptly to their cosmetical needs (Figure 3).

Many mechanisms including rebound dilatation of blood vessels, release of proinflammatory cytokines, and accumulation of nitric oxide thought to be responsible for the development of erythema, pruritus, and the burning sensation which produced by TCS misuse on the face [14]. The role of *Demodex folliculorum *in human dermatopathology and particularly in rosacea has remained controversial [5]. Bonnar et al. reported significantly increased mite densities in many types of rosacea including steroid-induced rosacea [15]. 

*Demodex *mites are also present on the skin of many healthy individuals so it has been suggested that the mite may have a pathogenic role only when it is present in high densities [16].

The average duration of TCS use required to produce the condition is 2–6 months, but it varies also according to the potency [7, 17].

Treatment of SDRR represents a challenge for the patients where it requires complete cessation of TCS use and avoidance of triggering factors. 

In the present work, all patients were given oral doxycycline 100 mg daily and topical combination of tacrolimus and tetracycline ointment for 2 months with a follow-up period every 2 weeks from starting the therapy; absolute discontinuation of TCS use was the most stressed factor for the patients. The patients also were advised to wash their faces only with plane water and to use sunscreen upon outdoor activity. All patients showed a good improvement in symptoms of SDRR within 4 weeks of 100 mg oral doxycycline along with topical tacrolimus and tetracyclin therapy.

All responders stopped topical therapy by the end of the 4th week and continued on another 4 weeks maintenance of oral doxycycline 100 mg daily. Telangiectatic patients showed only symptomatic response without disappearance of the telangiectasia. Thus, our experience agrees with other reports confirming the efficacy of doxycycline, tacrolimus, and topical tetracyclin therapy [7]. Calcineurin-inhibitors act through inhibition of the calcineurin-dependent signal transduction pathway, which is required for activation of CD4+ T-lymphocytes [18].

In conclusion, TCS misuse on the face is still a common condition where it is used as a miracle compound with believe that it will correct any imperfection on the face. However, after a variable duration, it will produce a red face which causes a lot of struggle for both patients and physicians.

## Figures and Tables

**Table 1:** Distribution of age in patient with SDRR using topical steroid.

Age distribution (years)	Number of patients (%)
11–20	6 (8%)
21–30	40 (53%)
31–40	25 (33%)
41–50	2 (2%)
51–60	2 (2%)

**Table 2:** Type of topical steroid used by SDRR patients.

Type of topical steroid used	Number of patients (%)
Clobetasol propionate	7 (9%)
Betamethasone valerate	5 (6%)
Both Clobetasol propionate and Betamethasone valerate	15 (20%)
Mixed with the cosmetics	48 (64%)

**Table 3:** Source of recommendation for topical steroids in patients with SRRD.

Source of prescription	Number of patients (%)
Beautician	26 (34%)
Self-prescription	20 (26%)
Pharmacy	18 (24%)
Dermatologist	7 (9%)
Relatives	2 (2%)
Friends	2 (2%)

**Table 4:** Purposes of using topical steroid on the face.

Purpose of topical steroid use	Number of patients (%)
Melasma	25 (33%)
Fairness	31 (41%)
Acne	9 (12%)
Freckles (blemishes)	5 (6%)
Actinic lichen planus	2 (2%)
Nonspecific dermatosis	3 (4%)

**Table 5:** Clinical findings in patients with SDRR using topical steroid.

Clinical findings	Number of patients (%)
Diffuse facial redness with hotness	70 (93%)
Dry facial skin	61 (81%)
Telangiectasia	58 (77%)
Rebound phenomenon	71 (94%)
Papulopustular lesions	30 (40%)
Papular rash without pustules	41 (54%)
Burning or itching	73 (97%)
Comedones	10 (13%)
Edema of the face	33 (44%)

**Table 6:** Triggering factors for SDRR patients.

Triggering factors	Number of patients (%)
Emotional stress	70 (93%)
Heat exposure	71 (94%)
Sun exposure	75 (100%)
Hair removal (threading)	67 (89%)
Spicy food	9 (12%)
Hot drinks	0 (0%)

## References

[1] Sulzberger MB, Witten VH, Yaffe SN (1951). Cortisone acetate administered orally in dermatologic therapy. *Archives of Dermatology and Syphilology*.

[2] Frumess GM, Lewis HM (1957). Light sensitive seborrheid. *Archives of Dermatology*.

[3] Mihan R, Ayres S (1964). Perioral dermatitis. *Archives of Dermatology*.

[4] Chen AYY, Zirwas MJ (2009). Steroid-induced rosacealike dermatitis: case report and review of the literature. *Cutis*.

[5] Leyden JJ, Thew M, Kligman AM (1974). Steroid rosacea. *Archives of Dermatology*.

[6] Ljubojeviæ S, Basta-Juzbašviæ A, Lipozenèiæ J (2002). Steroid dermatitis resembling rosacea: aetiopathogenesis and treatment. *Journal of the European Academy of Dermatology and Venereology*.

[7] Chen AYY, Zirwas MJ (2009). Steroid-Induced rosacealike dermatitis: case report and review of the literature. *Cutis*.

[8] Rathi S (2006). Abuse of topical steroid as cosmetic cream: a social background of steroid dermatitis. *Indian Journal of Dermatology*.

[9] Dubertret L (2002). Which steroids for the treatment of skin disorders on the face?. *Journal of the European Academy of Dermatology and Venereology*.

[10] Prawer SE, Katz HI (1990). Guidelines for using superpotent topical steroids. *The American Family Physician*.

[11] Lan NC, Karin M, Nguyen T (1984). Mechanisms of glucocorticoid hormone action. *Journal of Steroid Biochemistry*.

[12] Saraswat A, Lahiri K, Chatterjee M (2011). Topical corticosteroid abuse on the face: a prospective, multicenter study of dermatology outpatients. *Indian Journal of Dermatology, Venereology and Leprology*.

[13] Du Vivier A (1976). Tachyphylaxis to topically applied steroids. *Archives of Dermatology*.

[14] Rapaport MJ, Rapaport VH (2004). Serum nitric oxide levels in “red” patients: separating corticosteroid-addicted patients from those with chronic eczema. *Archives of Dermatology*.

[15] Bonnar E, Eustace P, Powell FC (1993). The Demodex mite population in rosacea. *Journal of the American Academy of Dermatology*.

[16] Erbağci Z, Özgöztaşi O (1998). The significance of Demodex folliculorum density in rosacea. *International Journal of Dermatology*.

[17] Sneddon I (1969). Adverse effect of topical fluorinated corticosteroids in rosacea. *British Medical Journal*.

[18] Goldman D (2001). Tacrolimus ointment for the treatment of steroid-induced rosacea: a preliminary report. *Journal of the American Academy of Dermatology*.

